# The Assessment of Multidimensional Clinical, Biological and Patient-Reported Outcomes to Evaluate the Efficacy of Add-On *Lactobacillus rhamnosus* GG Supplementation in Mild Ulcerative Colitis: A Randomized Pilot Trial

**DOI:** 10.3390/nu18091329

**Published:** 2026-04-23

**Authors:** Paola Maragno, Chiara Amoroso, Simone Conforti, Marco Michelon, Ivanna Honcharyuk, Clorinda Ciafardini, Daniele Noviello, Francesco Strati, Flavio Caprioli, Federica Facciotti, Maurizio Vecchi

**Affiliations:** 1 Gastroenterology and Endoscopy Unit, Fondazione IRCCS Ca’ Granda Ospedale Maggiore Policlinico, 20122 Milan, Italy; paola.maragno98@gmail.com (P.M.); chiara.amoroso@policlinico.mi.it (C.A.); francesco.conforti@policlinico.mi.it (S.C.); ivanna.honcharyuk@policlinico.mi.it (I.H.); clorinda.ciafardini@policlinico.mi.it (C.C.); francesco.strati@lsmu.lt (F.S.); flavio.caprioli@policlinico.mi.it (F.C.); 2 Department of Pathophysiology and Transplantation, Università degli Studi di Milano, 20122 Milan, Italy; marcomichelon8@gmail.com (M.M.); daniele.noviello@unimi.it (D.N.); 3 Institute for Digestive Research, Lithuanian University of Health Sciences, 44307 Kaunas, Lithuania; 4 Department of Biotechnology and Biosciences, University of Milano-Bicocca, 20126 Milan, Italy

**Keywords:** ulcerative colitis, *Lactobacillus rhamnosus* GG, adjunct therapy, fecal calprotectin

## Abstract

**Background:** Ulcerative colitis (UC) is a multifactorial disease characterized by aberrant mucosal immune activation in response to intestinal dysbiosis. Contemporary management strategies aim to target inflammation and microbiome alterations while reducing relapse risk. A multidimensional assessment integrating clinical, inflammatory, immune, and microbial endpoints may better capture therapeutic effects beyond symptom control. **Aims:** To evaluate whether supplementation with *Lactobacillus rhamnosus* GG co-formulated with vitamin D3 (Dicoflor IBD Immuno) as an adjunct to optimized mesalamine (5-ASA) is associated with coordinated changes across clinical and biological domains in mild-to-moderate UC, using a multidimensional assessment framework. **Methods:** This single-center, randomized, double-blind, placebo-controlled pilot trial was conducted at Fondazione Ca’ Granda IRCCS Policlinico di Milano between May 2022 and May 2024. Thirty-six patients with mild-to-moderate UC receiving optimized 5-ASA were randomized to LGG+VitD3 (ALD3) or placebo (AP) for 4 weeks. Clinical activity, health-related quality of life (HRQoL), fecal calprotectin, peripheral immune cell subsets, and gut microbiota composition were assessed at baseline and week 4. **Results:** Both 5-ASA-LGG+VitD3 (ALD3)- and 5-ASA-placebo (AP)-treated patients showed significant improvement in clinical activity and HRQoL, without between-group differences. A higher proportion of clinical responders was observed in the ALD3 group, although this was not statistically significant. LGG+VitD3-supplemented patients showed reduced fecal calprotectin levels and increased frequencies of IL-22-producing CD4^+^ T cells. Microbiome analysis revealed enrichment of short-chain fatty acid-producing taxa, including *Coprococcus* and *Fusicatenibacter*, in ALD3-treated patients. **Conclusions:** In patients with mild UC receiving optimized 5-ASA, LGG+VitD3 supplementation does not improve short-term clinical outcomes beyond placebo but is associated with favorable modulation of inflammatory, immune, and microbial parameters, supporting the relevance of multidimensional biological endpoints in adjunctive UC management.

## 1. Introduction

Ulcerative colitis (UC) is a chronic idiopathic inflammatory disorder of the colon characterized by a relapsing–remitting course. Together with Crohn’s disease (CD), it represents one of the two major forms of inflammatory bowel disease (IBD) [1]. UC pathogenesis results from complex interactions between genetic susceptibility, environmental factors, and intestinal microbiome alterations, leading to dysregulated mucosal immune responses [2,3,4].

In recent years, the concept of disease management in IBD has progressively expanded beyond the sole control of intestinal inflammation [5]. There is growing recognition that clinical symptoms, mucosal inflammation, immune dysregulation, gut microbiota composition, and patient well-being represent interconnected yet partially independent dimensions of disease activity [6,7]. Accordingly, clinical remission and quality-of-life improvement do not always parallel biological healing, highlighting the need for multidimensional evaluation strategies that integrate clinical, biochemical, immunological, and microbial endpoints.

Mesalamine (5-ASA) remains the standard therapy for mild-to-moderate UC, but combining anti-inflammatory and microbiota-modulating agents may improve disease control [8]. Indeed, patients with UC exhibit marked microbial dysbiosis, characterized by reduced microbial diversity, depletion of beneficial taxa with anti-inflammatory properties, and enrichment of pro-inflammatory species [9,10]. Reductions in Firmicutes and increases in Proteobacteria and Bacteroidetes have been associated with mucosal inflammation and impaired healing [4,11]. These alterations may persist even in patients with mild disease activity and contribute to ongoing low-grade inflammation. Thus, microbiome-targeted strategies have emerged as promising adjuncts to conventional therapy [12]. Probiotic supplementation can suppress pathogen colonization, reinforce epithelial barrier integrity, and modulate mucosal immune responses, although clinical efficacy has been variable across studies [13]. A recent Cochrane review suggested that probiotics, particularly when used in combination with standard therapies, may enhance remission induction [14]. Among probiotics, *Lactobacillus rhamnosus* GG (LGG; ATCC 53103) is one of the best-characterized strains, with an excellent safety profile and documented anti-inflammatory properties [15,16,17,18]. LGG has been shown to maintain remission and prevent pouchitis [19], though few studies have simultaneously evaluated its effects on clinical outcomes, immune function, and microbiome composition.

Vitamin D deficiency affects nearly half of patients with UC, and supplementation has been associated with reduced risk of disease relapse [20]. Vitamin D may contribute to intestinal homeostasis through modulation of mucosal immunity and activation of inflammasome-related pathways such as NLRP6 [21]. The combined administration of LGG and vitamin D3, therefore, represents a biologically plausible approach to support microbial balance and immune homeostasis in UC.

Within this evolving framework, early biological responses, such as changes in fecal calprotectin, immune cell profiles, and gut microbiota composition, may represent sensitive indicators of therapeutic impact, even in the absence of short-term clinical superiority over placebo [22,23,24]. This perspective is particularly relevant in mild UC, where optimized standard therapy and placebo effects can mask incremental clinical benefits [25].

Based on these considerations, we conducted a randomized, double-blind, placebo-controlled pilot study to explore the short-term, multidimensional effects of LGG co-formulated with vitamin D3 as an adjunct to optimized 5-ASA therapy in patients with mild-to-moderate UC. We aimed to assess coordinated changes across inflammatory biomarkers, immune responses, and gut microbiota composition, alongside conventional clinical and patient-reported outcomes, within a comprehensive and holistic disease management perspective.

## 2. Materials and Methods

### 2.1. Study Design

This study is a single-center, randomized, double-blind, placebo-controlled pilot trial designed to evaluate the short-term multidimensional effects of LGG+VitD3 as an adjunct to optimized 5-ASA therapy in patients with mild-to-moderate UC (trial registration number: ISRCTN12207718, 8 April 2026).

Patients were consecutively recruited from the IBD Outpatient Clinic at Policlinico Hospital, Milan (Italy), between 16 May 2022 and 29 May 2024 (Table 1). Participants were randomized (1:1) to receive either LGG Dicoflor IBD Immuno (*Lactobacillus rhamnosus* GG 1 × 10^10^ CFU plus vitamin D3 50 μg [2000 IU]) or matching placebo (one capsule daily) for 4 weeks, in addition to optimized 5-ASA therapy.

LGG and placebo capsules were prepared and provided by AG Pharma S.r.l.—Dicofarm SpA Group (Rome, Italy) in identical, unlabeled packaging. The trial was conducted in accordance with the Declaration of Helsinki and Good Clinical Practice guidelines, after approval by the Ethics Committee of IRCCS Fondazione Ca’ Granda Ospedale Maggiore Policlinico di Milano (study ID 2638; CE number 1172_2021, 14 December 2021). Written informed consent was obtained from all participants before inclusion.

Randomization was performed using REDCap (Research Electronic Data Capture) software (REDCap 13.4.11—2026 Vanderbilt University). All participants and study personnel remained blinded to treatment allocation throughout the study.

### 2.2. Participants

Inclusion criteria were: age 18–65 years, established diagnosis of ulcerative colitis, and mild-to-moderate disease activity defined by a baseline Partial Mayo Score (pMS) between 3 and 7, with a rectal bleeding subscore ≥ 1.

Exclusion criteria included: severe UC (pMS > 7), use of probiotics or systemic antibiotics within the previous four weeks, pregnancy or lactation, history of severe renal, hepatic, cardiac, or metabolic disease, and inability to comply with the study protocol. The only concomitant therapy allowed at the time of enrollment was oral or topical 5-ASA (suppositories or enemas). Prohibited medications included: other probiotics; antibiotics; corticosteroids (oral, parenteral, or rectal, including budesonide and beclomethasone dipropionate); immunosuppressants (6-mercaptopurine, azathioprine, methotrexate); and advanced therapies such as JAK inhibitors (tofacitinib, upadacitinib, filgotinib), anti-TNF agents (infliximab, adalimumab, golimumab), anti-IL-12/23 (Ustekinumab), and anti-integrins (vedolizumab). Substantial change in diet during the trial was considered a major protocol violation.

### 2.3. Procedures

After screening evaluation, eligible patients entered a two-week run-in period. At baseline (W0), patients enrolled signed the informed consent and 5-ASA therapy was optimized according to current guidelines (oral up to 4.8 g/day and topical up to 4 g/day).

Eligible patients were then randomized to receive LGG+VitD3 (ALD3) or placebo (AP) for 4 weeks. Treatment adherence was assessed by capsule count at each visit and was considered adequate if ≥80% of capsules were taken. Clinical evaluation, structured questionnaires, and biological sampling were performed at baseline (W0) and at week 4 (W4). Blood and stool samples were collected at W0 and W4 to assess fecal calprotectin levels, gut microbiome composition, and peripheral immune cell frequency and phenotype by multidimensional flow cytometry.

### 2.4. Study Endpoints

The primary endpoint was the change in health-related quality of life, assessed by the Inflammatory Bowel Disease Questionnaire (IBDQ), after 4 weeks of supplementation.

Secondary endpoints included: (i) change in clinical activity assessed by Partial Mayo Score, (ii) change in fecal calprotectin levels, (iii) changes in gut microbiome composition, and (iv) LGG-associated modulation of circulating immune cell subsets.

### 2.5. Structured Questionnaires

At W0 and W4, patients completed the following questionnaires: Short Form-36 (SF-36), Inflammatory Bowel Disease Questionnaire (IBDQ), Irritable Bowel Syndrome Severity Scoring System (IBS-SSS), and FACIT-Fatigue (FACIT-F). Detailed descriptions are provided in the Supplementary Materials [26].

### 2.6. Sample Size Calculation

Based on a recent meta-analysis [27], assuming a mean difference in IBDQ score of 17.5 (±5) in the placebo arm, a total sample size of 40 patients (20 per group) was estimated to detect a 30% difference between groups with 80% power at a significance level of 0.05 [16,28].

### 2.7. Fecal Calprotectin Measurement

Fecal calprotectin was measured on stool samples collected at baseline (W0) and at week 4 (W4). Samples were collected by patients using standardized collection devices and stored according to the manufacturer’s instructions until analysis. Quantitative determination of fecal calprotectin was performed using a particle-enhanced turbidimetric immunoassay on the SENTiFIT^®^ 270 analyzer (Sentinel Diagnostics, Milan, Italy), according to the manufacturer’s protocol. The assay employs monoclonal antibodies directed against human calprotectin and provides results expressed as μg/g of feces. The analytical measuring range of the assay is 10–2000 μg/g. Values exceeding the upper limit of detection were diluted and reanalyzed. Internal quality controls were run daily to ensure analytical accuracy and reproducibility. For descriptive and comparative analyses, fecal calprotectin values were categorized into three groups according to the interpretative ranges recommended for the CALiaGold^®^ assay on the SENTiFIT^®^ 270 analyzer: G1 < 70 μg/g (normal or no signs of intestinal inflammation), G2 70–355 μg/g (consider endoscopy), and G3 > 355 μg/g (perform endoscopy) [29].

### 2.8. Isolation of Peripheral Blood Mononuclear Cells

Peripheral blood mononuclear cells (PBMCs) were isolated from EDTA-anticoagulated whole blood collected at baseline (W0) and week 4 (W4). Samples were processed within 2 h of collection. PBMCs were separated by density gradient centrifugation using Ficoll–Paque™ PLUS (GE Healthcare, Chicago, IL, USA). After centrifugation (400× *g*, 30 min, room temperature, brake off), the mononuclear cell layer was collected, washed twice with phosphate-buffered saline, and resuspended in freezing medium (90% fetal bovine serum, 10% dimethyl sulfoxide). Cells were cryopreserved in liquid nitrogen until flow cytometry staining and analysis.

### 2.9. Cytofluorimetric Analysis

Cryopreserved PBMCs were thawed, washed, and stained with combinations of fluorochrome-conjugated monoclonal antibodies purchased from different vendors, as detailed in Supplementary Materials. Cells were first incubated with antibodies against surface markers for extracellular staining. Subsequently, cells were fixed and permeabilized using Cytofix/Cytoperm solution (BD Biosciences, Franklin Lakes, NJ, USA) and stained with antibodies specific for intracellular targets.

Samples were acquired on a FACSLyrics flow cytometer (BD Biosciences, Franklin Lakes, NJ, USA). Data were analyzed using FlowJo software (version 10.8; BD Biosciences). Doublets were excluded based on forward- and side-scatter parameters.

### 2.10. 16S rRNA Gene Sequencing and Data Analysis

The fecal microbiota was collected in TES buffer (50 mM Tris-HCl, pH 7.5, 10 mM NaCl, 10 mM EDTA) and stored at −80 °C. The bacterial DNA was extracted with a Genome DNA isolation kit (MP Biomedicals, Santa Ana, CA, USA) following the manufacturer’s instructions. 16S rRNA gene amplification, purification, library preparation, and paired-end sequencing using the Illumina MiSeq platform were performed as previously described [17]. We used the ZymoBIOMICS Microbial Community Standard II (Zymo Research, Irvine, CA, USA) as a mock community and negative controls to quantify and mitigate biases introduced during 16S rRNA gene sequencing procedures. Reads were pre-processed using the MICCA pipeline (v.1.7.2) [18,19]. Forward and reverse reads were merged, setting a minimum overlap length of 32 nucleotides and a maximum number of allowed mismatches equal to 8. Primers were trimmed using micca trim and reads with a length higher than 400 base pairs or an error rate higher than 1% were removed via micca filter. Filtered sequences were denoised using the UNOISE algorithm implemented in micca, removing all chimera sequences, to determine true biological sequences at the single-nucleotide resolution by generating ASVs. Bacterial ASVs were taxonomically classified using micca classify and the Ribosomal Database Project (RDP) Classifier v2.12, a naïve Bayesian classifier [20]. Multiple sequence alignment of 16S sequences was performed using the Nearest Alignment Space Termination (NAST) algorithm [21] implemented by micca msa with the template alignment clustered at 97% similarity of the Greengenes core set [22]. Phylogenetic trees were inferred using micca tree. The number of reads per sample varied between 16,722 and 158,549; sampling heterogeneity was reduced by rarefying samples, using micca tablerare, at a rarefaction threshold of 16,722 reads per sample, equivalent to the depth of the less abundant sample. Eventually, micca tabletotax was used to summarize communities by their taxonomic composition. All the subsequent analyses were performed in the R environment using the phyloseq R package (v1.48.0) [23]. The mean relative abundances across samples of these genera were computed separately for non-malnourished and moderately or severely malnourished patients, both for healthy and tumor tissues, and plotted using ggplot2 R package (v3.5.1) [24]. Alpha (within-sample richness evaluated by computing the observed richness and the Shannon index) and beta-diversity (between-sample dissimilarity evaluated by computing the Bray–Curtis distance) estimates were computed using the phyloseq R package. Alpha diversity values were tested using the wilcox_test function of the Rstatix R package (v0.7.2) [25] considering separately paired and unpaired samples. The permutational multivariate analysis of variance (PERMANOVA) test was performed using the adonis function in the vegan R package (v2.6.6.1) [26,27] with 999 permutations to test beta diversity values. The differential abundance of the different ASVs—comparing non-malnourished and moderately or severely malnourished patients, considering healthy and tumor samples separately—was tested using the DESeq2 R package (v1.44.0) [27] on the non-rarefied data. *p*-values were false-discovery rate-corrected using the Benjamini–Hochberg procedure implemented in DESeq2. The results were represented in a volcano plot generated by the ggplot2 R package; only the ASVs with a nominal *p*-value < 0.05 were colored, and the name was reported only for the subset with a nominal *p*-value < 0.05 and an absolute log2FC > 1. Spearman’s correlation test between the abundances of the ASVs differently enriched between the two groups of patients—separately in healthy and tumor samples—and the abundances of the different biochemical molecules and LMPCs were computed using corr.test function from the psych R package (v2.5.3). The resulting correlation values were represented in a heatmap using the pheatmap R package (v1.0.12) [28].

### 2.11. Statistical Analysis

The data analysis was performed according to the Intention-to-Treat (ITT) principle, which included all patients initially assigned to the treatment groups, regardless of adherence to the treatment or dropouts during the study.

All statistical analyses were performed in R environment v4.4.1 and GraphPad Prism v10.2.3. Parametric *t*-test was used as well as different non-parametric tests like Chi-squared test with Yates’ continuity correction, Fisher’s exact test, PERMANOVA, Wilcoxon test for paired samples, and Mann–Whitney Test for unpaired samples. The *p*-values were corrected using the Benjamini–Hochberg correction.

## 3. Results

### 3.1. Demographic Characteristics of the Population

Between 16 May 2022 and 29 May 2024, 50 patients with UC were screened for eligibility. Ten eligible patients declined participation, two were excluded because they did not meet disease severity criteria, and two withdrew before randomization. In total, 36 patients with mild-to-moderate UC were ultimately enrolled and randomized (Table 1 and Figure S1). In addition to optimized 5-ASA, participants were randomly divided into two groups: 18 were additionally supplemented with the probiotic LGG Dicoflor IBD Immuno (ALD3), while the other 18 were given a placebo for a duration of 4 weeks, 1 anonymized capsule per day (AP). At baseline, the median Partial Mayo Score was 4 (IQR 3–5). No significant differences were observed between treatment groups with respect to demographic or clinical characteristics (Table 1). Of the entire cohort, 69.4% (*n* = 25) were females, with a median age of 48.5 years (IQR: 35–59.25). The median disease duration after diagnosis was 10 years (IQR: 5.75–15), with 41.7% of patients affected by the disease for more than 12 years. At baseline, according to the Montreal Classification, 47.2% (*n* = 17) had ulcerative proctitis (E1), 44.5% (*n* = 16) had left-sided ulcerative colitis (E2), and only 8.3% (*n* = 3) had extensive ulcerative colitis (E3). Even though all three patients with extensive ulcerative colitis (E3–4) were randomly assigned to the ALD3 group, no differences were observed between groups in terms of disease extent (*p* = 0.29). The median BMI was 23.31 (IQR: 20.68–26.14), with 66.7% of patients classified as having a normal weight. A history of previous corticosteroid use was reported by 55.6% (*n* = 20) of the patients.

### 3.2. HRQoL Is Not Improved in ALD3 Compared with AP Patients, but ALD3 Supplementation Shows a Trend Toward Higher Clinical Response

**Figure 1:**
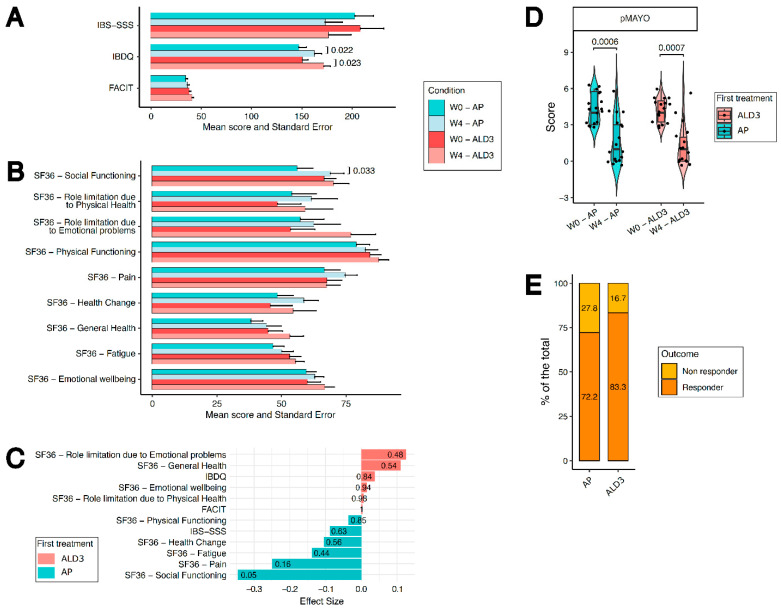
Clinical and patient-reported outcomes in ALD3- and AP-treated patients after 4 weeks. Score variation in the IBDQ, FACIT-F and IBS-SSS (**A**) and SF36 (**B**) at enrollment (W0), at the end of supplementation (W4) in AP (placebo)- (blue graphs) or ALD3 (LGG+VitD3)-treated subjects (pink graphs). Statistical analysis has been performed by Wilcoxon–Mann–Whitney test, two-tailed. *p* < 0.05 was considered statistically significant. (**C**) Effect size of changes from baseline to week 4 for SF-36 domains in ALD3- and AP-treated patients. Statistical analysis has been performed by Mann–Whitney test, two-tailed. (**D**) Score variation in the pMS at enrollment (W0), at the end of supplementation (W4) in AP- (blue graphs) or ALD3-treated subjects (pink graphs). Statistical analysis has been performed by Wilcoxon–Mann–Whitney test, two-tailed. *p* < 0.05 was considered statistically significant. (**E**) Percentage of clinical responders at W4 in the AP or ALD3 groups. Statistical difference was evaluated with Fisher’s exact test (*p* = 0.69). Statistical analysis has been performed by Wilcoxon–Mann–Whitney test, two-tailed. *p* < 0.05 was considered statistically significant.

The primary endpoint of the study was the comparison of health-related quality of life (HRQoL) between ALD3- and AP-treated patients after 4 weeks of supplementation, assessed using validated patient-reported outcome measures including the Inflammatory Bowel Disease Questionnaire (IBDQ), Short Form-36 (SF-36), FACIT-Fatigue (FACIT-F), and IBS Severity Scoring System (IBS-SSS). Both ALD3- and AP-treated patients showed a significant improvement in IBDQ scores from baseline to week 4 (Figure 1A). A significant improvement in the SF-36 questionnaire was observed only in the Social Functioning domain within the placebo group (Figure 1B). However, no significant between-group differences were detected across the evaluated HRQoL measures, and ALD3 supplementation did not demonstrate overall superiority compared with placebo (Figure 1A–C). Exploratory analyses of HRQoL at week 16 are reported in Figure S2 and did not reveal significant between-group differences.

Consistently, ALD3 and AP patients showed a significant reduction in Partial Mayo Score (pMS) at week 4 compared with baseline (Figure 1D). When patients were classified according to clinical response (defined as a reduction ≥ 2 points in pMS and improvement in rectal bleeding subscore), a higher proportion of clinical responders was observed among ALD3-treated patients compared with AP-treated patients (83.3% vs. 72.2%), although this difference did not reach statistical significance (Figure 1E). Clinical responders in both arms showed significant improvement in all health-related domains evaluated (Figure S3A). No difference was observed among clinical responders in terms of age and BMI, but women tended to respond less to ALD3 supplementation (Figure S3B–D).

Overall, these findings indicate that short-term LGG+VitD3 supplementation does not confer additional benefit on patient-reported outcomes beyond placebo in patients receiving optimized 5-ASA therapy.

### 3.3. LGG+VitD3-Supplemented Patients Show Modulation of Fecal Calprotectin Levels and Immune Cell Subsets

**Figure 2:**
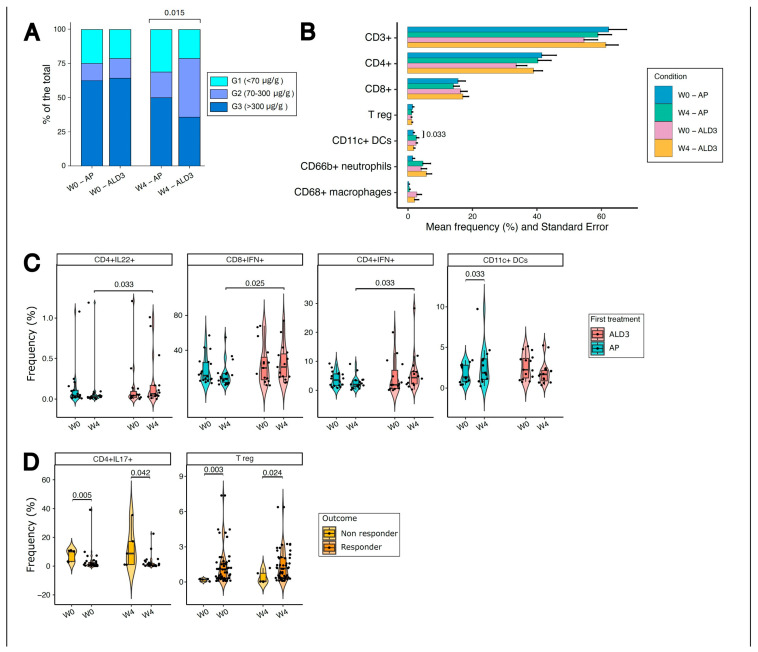
Changes in fecal calprotectin categories and circulating immune cell subsets after 4 weeks of ALD3 supplementation. (**A**) Percentage of patients at baseline and at W4 in the AP (placebo) or ALD3 (LGG+VitD3) arms, divided into 3 groups (G1, G2, G3) according to fecal calprotectin levels. Statistical difference at W4 was evaluated with Chi-squared test with Yates’ continuity correction (*p* = 0.015). (**B**) Mean frequencies (±SEM) of circulating immune cell subsets at baseline (W0) and week 4 (W4) in AP- and ALD3-treated patients, including total T cells (CD3^+^), CD4^+^ and CD8^+^ T cells, regulatory T cells (Treg), CD11c^+^ dendritic cells, CD66b^+^ neutrophils, and CD68^+^ macrophages. (**C**) Frequency of cytokine-producing T-cell subsets among AP and ALD3 groups at baseline and 4 weeks post-supplementation and (**D**) among clinical responders and not responders. Statistical analysis has been performed by Wilcoxon–Mann–Whitney test, two-tailed. *p* < 0.05 was considered statistically significant.

Fecal calprotectin (FC), a non-invasive biomarker of intestinal inflammation, was used to assess changes in inflammatory activity. FC values were categorized according to the interpretative ranges recommended for the CALiaGold^®^ assay on the SENTiFIT^®^ 270 analyzer: G1 < 70 μg/g (normal or no signs of intestinal inflammation), G2 70–355 μg/g (suggestive of moderate inflammation), and G3 > 355 μg/g (associated with significant inflammation) [29]. At baseline, the distribution of patients across these categories was comparable between ALD3- and AP-treated groups. After 4 weeks of supplementation, a higher proportion of ALD3 patients shifted toward lower fecal calprotectin categories (G1 and G2) compared with placebo (Figure 2A; *p* = 0.015), suggesting a potential reduction in intestinal inflammatory burden. However, these findings should be interpreted with caution given the limited sample size.

Flow cytometric analysis of circulating PBMCs revealed selective modulation of both adaptive and innate immune cell compartments in ALD3-treated patients. Frequencies of circulating pro-inflammatory T cells, Treg cells, macrophages and neutrophils were similar between AP- and ALD3-treated patients (Figure 2B). However, LGG+VitD3 supplementation was associated with a higher frequency of IL-22-producing CD4^+^ T cells at week 4, a subset implicated in epithelial barrier repair. Modest increases in IFN-γ-producing CD4^+^ and CD8^+^ T cells were also observed in the ALD3 group, whereas dendritic cell frequencies were relatively higher in AP-treated patients (Figure 2C). In addition, when patients were stratified according to clinical response, responders exhibited a higher frequencies of regulatory T cells and lower frequencies of Th17 cells compared with non-responders, irrespective of treatment allocation (Figure 2D).

Together, these data suggest that LGG+VitD3 supplementation in addition to 5-ASA is associated with coordinated early changes in intestinal inflammation and systemic immune profiles, supporting a biological effect that is not fully reflected by short-term clinical or HRQoL outcomes.

### 3.4. LGG+VitD3 Supplementation in Addition to Mesalamine Is Associated with Shifts in Gut Microbiota Composition at Week 4

The intestinal microbiome plays a critical role in the pathogenesis of UC [30], and dysbiosis is a hallmark of the disease. Given the modulation of fecal calprotectin and immune subsets observed in ALD3 patients, we next investigated whether changes in microbiota composition occurred after 4 weeks of treatment.

**Figure 3:**
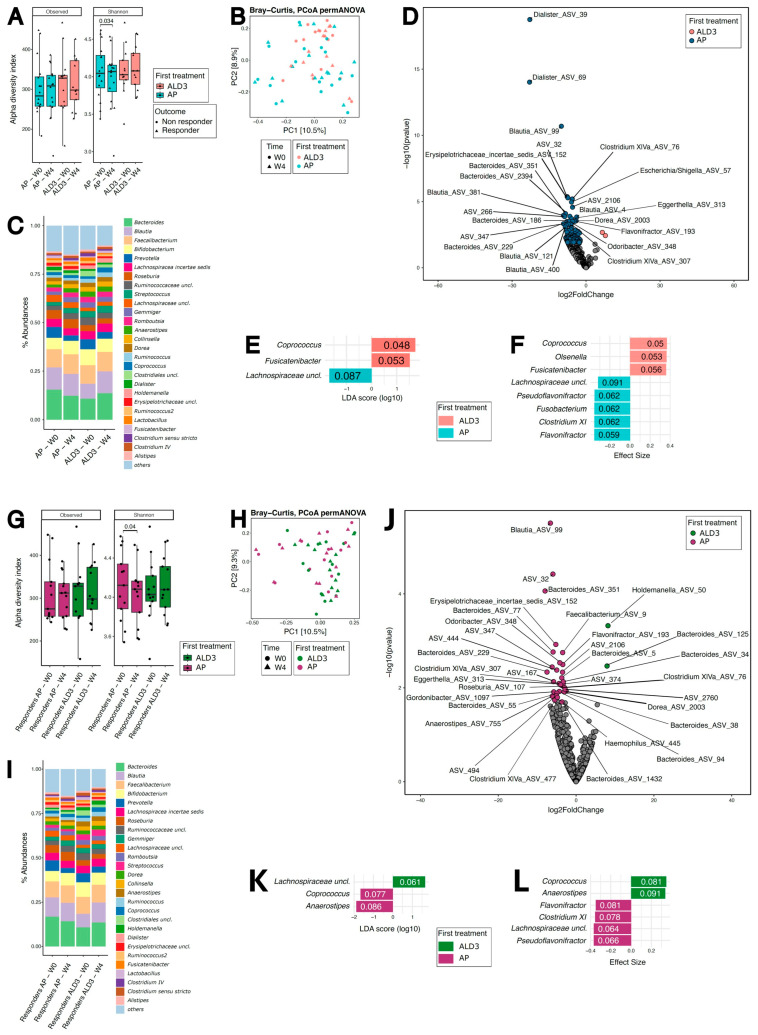
LGG-VitD3 supplementation as add-on therapy to mesalamine modulates gut microbiota composition. (**A**,**B**) Alpha ((**A**), observed and Shannon index) and beta ((**B**), Bray–Curtis distance) diversity of AP (placebo; blue symbols)- and ALD3-treated (LGG+VitD3; pink symbols) patients at baseline and W4. Grey dots represent individual data points. Statistical analysis on alpha diversity scores has been performed by Wilcoxon–Mann–Whitney test, two-tailed. *p* < 0.05 was considered statistically significant; statistical analysis on beta diversity scores has been performed by PERMANOVA (not significant). (**C**) Mean relative abundance at W0 and W4 of the most abundant bacterial taxa in AP and ALD3-patients. (**D**) Volcano plot of the most significantly enriched taxa after supplementation (W4) between AP (blue)- and ALD3 (pink)-treated patients. Colored points are those with absolute log2FC > 1 and FDR < 0.05; name is reported for ASVs with absolute log2FC > 1 and FDR < 0.01. (**E**,**F**) LDA (**E**) and effect size (**F**) of taxa enrichment at W4 between ALD3 and AP groups. Statistical analysis has been performed by LEfSe and Mann–Whitney test, two-tailed. (**G**,**H**) Alpha ((**G**), observed and Shannon index) and beta ((**H**), Bray–Curtis distance) diversity between clinical responders belonging to the AP group (violet) and clinical responders of the ALD3 group (green). Statistical analysis on alpha diversity scores has been performed by Wilcoxon–Mann–Whitney test, two-tailed. *p* < 0.05 was considered statistically significant. Statistical analysis on beta diversity scores has been performed by PERMANOVA (not significant). (**I**) Mean relative abundance at W0 and W4 of the most abundant bacterial taxa between clinical responders among ALD3 and AP patients. (**J**) Volcano plot of the most significantly enriched taxa after supplementation (W4) between clinical responders among AP (violet)- and ALD3 (green)-treated patients. Colored points are those with absolute log2FC > 1 and *p* < 0.02; name is reported for ASVs with absolute log2FC > 1 and *p* < 0.02. (**K**,**L**) LDA (**K**) and effect size (**L**) of taxa enrichment at W4 between ALD3 and AP groups. Statistical analysis has been performed by LEfSe and Mann–Whitney test, two-tailed.

At baseline, no significant differences in alpha or beta diversity were observed between AP- and ALD3-treated patients (Figure 3A–C). However, after 4 weeks of supplementation, ALD3-treated patients exhibited an increase in the abundance of beneficial, short-chain fatty acid (SCFA)-producing taxa, including *Coprococcus* and *Fusicatenibacter* (Figure 3D–F). In contrast, AP-treated patients showed a relative enrichment of pro-inflammatory taxa, including *Dialister* and *Escherichia*/*Shigella*, which have been associated with UC-related inflammation (Figure 3D–F). Clinical responders in both groups demonstrated higher alpha diversity compared with non-responders (Figure S4A–C) and showed an enrichment of *Akkermansia* as compared with non-responders (Figure S4D–F). Interestingly, no major differences were observed between the groups in terms of beta diversity (Figure 3G–I), but ALD3-treated patients tended to show a greater proportion of health-associated taxa. Indeed, ALD3-treated clinical responders exhibited further enrichment of beneficial SCFA-producing bacteria, such as *Holdemanella* and *Coprococcus*, compared to non-responders (Figure 3J–L).

These findings indicate that LGG+VitD3 supplementation in addition to 5-ASA leads to a shift toward a more favorable microbiota profile, with enrichment of health-associated taxa and a potential reduction in pro-inflammatory bacteria, which were enriched in the placebo group, highlighting the broader benefits of this intervention in UC management.

## 4. Discussion

In this pilot study, we explored the effects of *Lactobacillus rhamnosus* GG (LGG) co-formulated with vitamin D3 (VitD3) as an adjunct to optimized 5-ASA therapy in patients with mild-to-moderate ulcerative colitis (UC). While our results revealed no significant improvements in clinical outcomes or health-related quality of life (HRQoL) between the ALD3 and AP groups, the add-on supplementation of LGG+VitD3 was connected to favorable changes in inflammatory biomarkers, immune profiles, and gut microbiota composition. Exploratory longer-term HRQoL data (week 16) did not show clear between-group differences, supporting the consistency of short-term observations. These findings provide a nuanced perspective on the role of adjunct therapies in UC management, underscoring the importance of a multidimensional approach to disease evaluation, which extends beyond traditional clinical outcomes.

Our study aligns with previous reports suggesting that short-term interventions may not always yield direct clinical improvements, especially in patients with mild UC [31,32,33]. This variability in outcomes is not uncommon in studies of probiotics, where biological effects on immune modulation and microbiome composition may not immediately translate into measurable clinical changes [14,34]. Both groups in our study demonstrated significant improvements in clinical activity scores, but the lack of significant differences between the two groups in clinical response and HRQoL scores is consistent with the fact that patients are receiving standardized therapies like 5-ASA [27]. The beneficial effects of probiotics in these contexts may therefore be better appreciated through quantitative rather than qualitative biomarkers, including fecal calprotectin, microbiome composition, and immune system parameters. In this context, previous probiotic RCTs may have failed to provide strong signals as they may have been masked by the strong clinical response, not accompanied by a detailed measurement of coordinated changes across inflammatory biomarkers, immune responses, and gut microbiota composition. An important aspect to consider is the potential dissociation between early biological responses and short-term clinical outcomes. In IBD, clinical symptoms and patient-reported measures do not always parallel changes in inflammatory activity or immune modulation, particularly over short observation periods [22,23,35]. Therefore, the modulation of fecal calprotectin, immune cell subsets, and microbiota composition observed in our study may represent early biological effects that are not immediately reflected in clinical endpoints. This temporal disconnection has been reported in previous studies and highlights the need for longer-term evaluation to determine whether early biological changes translate into sustained clinical benefit [22,23,35].

A key strength of our study lies in the use of optimized mesalamine therapy as background treatment. In contrast, earlier randomized trials investigating probiotics in UC, such as the widely cited study by Kruis et al. [36], compared probiotic formulations with relatively low-dose mesalamine regimens, which may have functioned as suboptimal or near-placebo comparators. In our study, all patients received optimized 5-ASA therapy according to current clinical practice, providing a more stringent and clinically relevant framework to evaluate the incremental benefit of adjunctive interventions. While this design may have reduced the ability to detect additional clinical effects of LGG+VitD3, it strengthens the translational relevance of our findings by reflecting real-world therapeutic conditions.

Despite the absence of differences in clinical endpoints, we observed a significant modulation of fecal calprotectin levels in the ALD3 group compared to the AP. Fecal calprotectin is a well-established biomarker of intestinal inflammation, and its reduction suggests a potential anti-inflammatory effect of LGG supplementation, which has been previously documented in studies of probiotics in IBD [37,38]. Moreover, immune profiling revealed selective modulation of immune subsets, including an increased frequency of IL-22-producing CD4^+^ T cells, a subset involved in epithelial barrier repair [39]. However, modulation of IL-22-producing T cells over short timeframes may reflect early and potentially transient immune responses, which are not necessarily indicative of sustained mucosal healing. These immune changes, although not directly reflected in clinical symptom improvement, suggest early biological effects of LGG+VitD3 that could become more pronounced with longer-term treatment, echoing findings from other microbiome-based interventions in UC [12].

Microbiome analysis also revealed a shift toward a more beneficial gut microbiota composition with add-on ALD3 supplementation, including an increased relative abundance of taxa commonly associated with short-chain fatty acid (SCFA) production like *Coprococcus* and *Fusicatenibacter*. This finding is consistent with studies showing that probiotics can positively influence microbial diversity in UC, potentially reducing inflammation by restoring beneficial microbial communities [40,41]. The increase in SCFA-producing bacteria suggests a potential mechanism by which ALD3 supplementation in addition to 5-ASA may exert its anti-inflammatory effects, aligning with emerging evidence on the role of gut microbiota in IBD pathogenesis [4,30]. It is important to note that the microbiome findings presented here are based on taxonomic profiling and therefore remain correlative. Although the enrichment of taxa commonly associated with SCFA production suggests a potentially beneficial microbial shift, no direct functional measurements were performed. Future studies integrating metabolomic analyses, such as fecal SCFA quantification, together with longitudinal microbiome assessments, will be essential to establish the mechanistic relevance of these observations.

A central message of our study is the need for a holistic approach to UC management. Traditional clinical measures, such as the Mayo score, do not always fully capture the complex nature of the disease, which involves the interplay between immune dysregulation, microbiome alterations, and patient well-being [35]. Our results support the idea that a comprehensive disease management approach should integrate clinical, biochemical, immunological, and microbial endpoints. This multidimensional framework would enable clinicians to better assess the therapeutic potential of adjunct therapies, such as probiotics, beyond just clinical symptom relief.

The limitations of our study are notable. As a pilot trial, the sample size was small, and the treatment duration was short, which inherently limits the generalizability of our findings. The lack of significant clinical improvement may reflect the small sample size and the limited statistical power of the study. Additionally, the short-term nature of the intervention means that we cannot assess whether the observed biological effects would lead to long-term clinical benefits. Furthermore, as this was a pilot study, it was not powered to detect small clinical differences, and future trials with larger cohorts and extended follow-up periods are necessary to confirm these findings. Importantly, the limited sample size substantially restricts the statistical power to detect between-group differences, particularly for clinical endpoints. Therefore, several of the observed differences across inflammatory, immunological, and microbiome parameters should be interpreted as exploratory trends rather than definitive effects. These findings should be considered hypothesis-generating and require validation in larger, adequately powered studies. Another important limitation of the present study is the combined administration of LGG and vitamin D3, which does not allow the individual contribution of each component to be disentangled. Although this combination was selected based on their potentially complementary effects on immune modulation and microbiota homeostasis [42,43], the current study design does not permit attribution of the observed biological changes to either LGG or vitamin D3 alone. Future studies including separate intervention arms will be necessary to clarify the individual and synergistic effects of these components.

## 5. Conclusions

In conclusion, our data suggest that ALD3 supplementation as add-on therapy to mesalamine, while not improving short-term clinical outcomes in patients with mild UC, may be associated with changes in inflammatory biomarkers, immune responses, and microbiota composition. These findings support the relevance of a multidimensional approach to UC management that considers not only clinical symptoms but also the underlying biological processes driving the disease.

From a translational perspective, it will be important to determine whether these early biological changes translate into sustained clinical benefits over longer follow-up periods. Accordingly, larger studies with extended follow-up and integrated clinical and multi-omics analyses will be essential to assess the durability, functional relevance, and therapeutic potential of these findings.

## Figures and Tables

**Table 1:** Demographics and Clinical Characteristics of the Patients Included in the Study. For continuous variables, the median and first and third quartiles are reported; for categorical variables, the percentage with respect to the total per group is reported. To test the difference between AP (placebo)- and ALD3 (LGG+VitD3)-treated patients, the nominal *p*-value by Mann–Whitney test was computed for continuous variables; the nominal *p*-value by Chi-squared test with Yates’ continuity correction or by Fisher’s exact test was computed for categorical variables.

Clinical Parameter	Total (*n* = 36)	AP (*n* = 18)	ALD3 (*n* = 18)	*p*-Value
Male/female, *n* (%)	11/25 (30.6)	5/13 (27.8)	6/12 (33.3)	>0.9
Age	48.5 (35; 59.25)	48.5 (39; 55)	48 (34; 59.75)	0.72
Duration after diagnosis (years)	10 (5.75; 15)	12.5 (5.5; 16.5)	8.5 (6; 14.75)	0.42
<3	3 (8.3)	1 (5.6)	2 (11.1)	>0.9
3–6	9 (25)	4 (22.2)	5 (27.8)	>0.9
7–9	5 (13.9)	3 (16.7)	2 (11.1)	>0.9
10–12	4 (11.1)	1 (5.5)	3 (16.7)	0.6
>12	15 (41.7)	9 (50)	6 (33.3)	0.5
Disease extent according to Montreal classification				
Ulcerative proctitis (E1)	17 (47.2)	9 (50)	8 (44.4)	>0.9
Left-sided ulcerative colitis (E2)	16 (44.5)	9 (50)	7 (38.9)	0.74
Extensive ulcerative colitis (E3)	3 (8.3)	0	3 (16.7)	>0.9
Body mass index BMI	23.31 (20.68; 26.14)	23.91 (20.61; 26.7)	23.18 (20.78; 24.24)	0.65
<18.5	1 (2.8)	1 (5.5)	0	>0.9
18.5 and <25	24 (66.7)	10 (55.6)	14 (77.8)	0.29
25≤ and <30	9 (25)	6 (33.3)	3 (16.7)	0.44
30≤ and <35	2 (5.5)	1 (5.6)	1 (5.5)	>0.9
≥35	0	0	0	>0.9
History of previous steroid medication	20 (55.6)	11 (61.1)	9 (50)	0.74
Concomitant medication	36 (100)	18 (100)	18 (100)	>0.9
5-ASA, per-oral	36 (100)	18 (100)	18 (100)	>0.9
5-ASA, topical agent	36 (100)	18 (100)	18 (100)	>0.9

## Data Availability

The 16S rRNA gene sequencing data generated in this study have been deposited in the European Nucleotide Archive (ENA) under accession number PRJEB91943 (secondary accession: ERP174873). Clinical and immunological data are available from the corresponding author upon reasonable request, subject to ethical and privacy restrictions.

## Supplementary Materials

The following supporting information can be downloaded at https://www.mdpi.com/article/10.3390/nu18091329/s1: Figure S1: CONSORT Flow Diagram; Figure S2: Exploratory analysis of HRQoL and symptom questionnaires at week 16 (W16); Figure S3: Changes in patient-reported outcomes and clinical characteristics at week 4 stratified by response status; Figure S4: Microbiome analyses at the end of W4; Table S1: Antibodies used for multiparametric FACS analysis.

## Abbreviations

The following abbreviations are used in this manuscript:

5-ASA	5-Aminosalicylic Acid (Mesalamine)
ALD3	5-ASA + *Lactobacillus rhamnosus* GG + Vitamin D3 group
AP	5-ASA + Placebo Group
CD	Crohn’s Disease
FACIT-F	Functional Assessment of Chronic Illness Therapy–Fatigue
FC	Fecal Calprotectin
HRQoL	Health-Related Quality of Life
IBD	Inflammatory Bowel Disease
IBDQ	Inflammatory Bowel Disease Questionnaire
IBS-SSS	Irritable Bowel Syndrome Severity Scoring System
IFN-γ	Interferon gamma
IL	Interleukin
ITT	Intention-to-Treat
LGG	*Lactobacillus rhamnosus* GG
PBMCs	Peripheral Blood Mononuclear Cells
pMS	Partial Mayo Score
RDP	Ribosomal Database Project
RCT	Randomized Controlled Trial
SCFA	Short-Chain Fatty Acid
SF-36	Short Form-36 Health Survey
UC	Ulcerative colitis
VitD3	Vitamin D3
W0	Week 0 (Baseline)
W4	Week 4
