# Supplementary material for: The Assessment of Multidimensional Clinical, Biological and Patient-Reported Outcomes to Evaluate the Efficacy of Add-On Lactobacillus rhamnosus GG Supplementation in Mild Ulcerative Colitis: A Randomized Pilot Trial
Source: Nutrients. 2026 Apr 23;18(9):1329. doi: 10.3390/nu18091329 (PMC13165221; doi:10.3390/nu18091329)
Supplement: Supplementary file 1 [file nutrients-18-01329-s001.zip › nutrients-4201540-supplementary.pdf]

Assessment of multidimensional clinical, biological and patient-reported outcomes to evaluate the efficacy of add-on *Lactobacillus rhamnosus* GG Supplementation in Mild Ulcerative Colitis: A Randomized Pilot Trial

Paola Maragno[1*], Chiara Amoroso[1*], Simone Conforti[1*], Marco Michielon[1,2], Ivanna Honcharyuk [1], Clorinda Ciafardini [1], Daniele Noviello[1,2], Francesco Strati[1], Flavio Caprioli[1,2], Federica Facciotti[3§], Maurizio Vecchi[1,2§]

Supplementary material

Supplementary Methods

pMAYO scoring

Since endoscopy could not be performed at every visit, the partial Mayo Score (pMayo) was implemented. The Mayo Score includes four components: stool frequency (SF), rectal bleeding (RB), endoscopic appearance of the mucosa (EM), and a Physician's Global Assessment (PGA). Each component was scored on a scale from 0 to 3, resulting in a maximum total score of 12. i) Stool frequency: 0 = Normal, 1 = 1-2 stools/day more than normal, 2 = 3-4 stools/day more than normal, 3 = ≥5 stools/day more than normal; ii) Rectal bleeding: 0 = None, 1 = Visible blood with stool less than half the time, 2 = Visible blood with stool half of the time or more, 3 = Blood alone passed; iii) Endoscopic appearance: 0 = Normal or inactive disease, 1 = Mild disease (erythema, decreased vascular pattern, mild friability), 2 = Moderate disease (marked erythema, absent vascular pattern, friability, erosions), 3 = Severe disease (spontaneous bleeding, ulceration); iv) Physician's Global Assessment: 0 = Normal, 1 = Mild, 2= Moderate, 3= Severe. Score Decoding Partial Mayo Score: ≤ 2 = Remission; 3-4 = Mild activity; 5-7 = Moderate activity and > 7 = Severe activity.

Questionnaires

All items use 7-point Likert-type scales for capturing symptom-related experiences over the previous 2 weeks, with 1 indicating the highest symptom frequency/severity and 7 indicating the lowest symptom frequency/severity. Higher domain and total scores indicate better health-related quality of life (HRQoL).

<u>Short Form-36 (SF)-36:</u> (SF)-36 consists of 36 items that measure eight different domains: physical functioning, role limitations due to physical problems, bodily pain, general health perceptions,

vitality, social functioning, role limitations due to emotional problems, and mental health. Scoring the (SF)-36 involves transforming raw item responses into standardized scores ranging from 0 to 100 for each domain. Higher scores indicate better health-related quality of life in the respective domain.

Irritable Bowel Syndrome Symptom Severity Score (IBS-SSS) is a 5-item questionnaire that assesses the severity of IBS symptoms like abdominal pain, bloating, dissatisfaction with bowel habits, and quality of life interference over the past 10 days. Each of the five items is scored from 0 to 100, resulting in a total score from 0 to 500, where higher scores indicate greater severity. Scores are categorized to define severity: mild (75-174), moderate (175-299) severe (300-500).

FACIT-Fatigue Scale: is a short, 13-item, easy to administer tool that measures an individual's level of fatigue during their usual daily activities over the past week. The level of fatigue is measured on a four-point Likert scale (4 = not at all fatigued to 0 = very much fatigued) (Webster et al., 2003).

Inflammatory Bowel Disease Questionnaire (IBDQ): is a 32-item survey that measures quality of life in patients with inflammatory bowel disease. The total score ranges from 32 to 224, with higher scores indicating a better quality of life. The questionnaire is divided into four domains: bowel symptoms, systemic symptoms, social function, and emotional functioning, and a score of 170 or higher may indicate clinical remission.

Clinical data analysis

Chi-squared test with Yates' continuity correction or Fisher's exact test and Mann-Whitney test were used to evaluate differences in the demographics and clinical characteristics between LGG-treated and placebo-treated patients. Chi-squared test with Yates' continuity correction was computed to evaluate whether the number of responders and non-responders in LGG-treated (ALD3) and placebo (AP)-treated groups was significantly different at W4. The distributions of the scores obtained by the patients in the questionnaires were represented as violin plots or barplots and the differences in these distributions between the different conditions were tested using wilcox_test function of rstatix R package (v0.7.2) (https://rpkgs.datanovia.com/rstatix/] considering separately paired and unpaired samples. The scores of IBS-SSS and pMAYO questionnaires were transformed as the maximum score of that questionnaire minus the actual score so that all the questionnaires have the same trend: the higher the score the better the condition. For each patient and each questionnaire, the difference between the score obtained at two different time points was computed in R and converted into percentage with respect to the maximum score of that questionnaire. The difference between the percentage variation obtained by patients of the different groups under evaluation was tested using wilcox_test and wilcox_effsize functions of rstatix R package (v0.7.2). Nominal p-values and effect sizes were represented as a barplot. The distribution of the scores of each questionnaire was computed

at different time points also dividing patients based on the combination of treatments given at the randomization. Differences between these distributions were evaluated using t_test function of rstatix R package (v0.7.2).

Supplementary Table S1: Antibodies used for multiparametric FACS analysis.

	Clone
CD3	UCHT1
CD4	A161A1
CD8	SK1
IL10	JES3-19F1
IFNγ	4S.B3
IL17	eBio64DEC17
IL22	2G12A41
CD11c	3.9
CD66b	G10F5
CD68	eBIoY1/82A

Supplementary Figures

Supplementary Figure S1: CONSORT Flow Diagram.

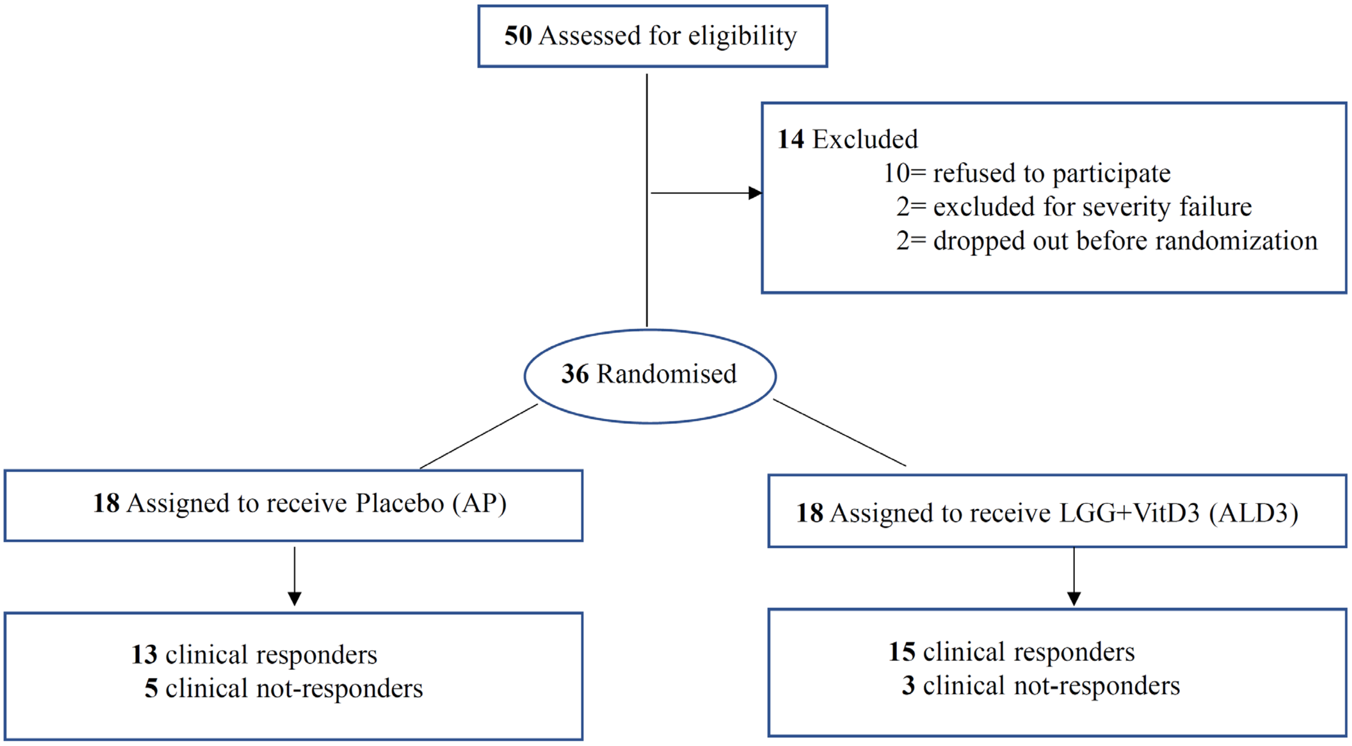

Supplementary Figure S2

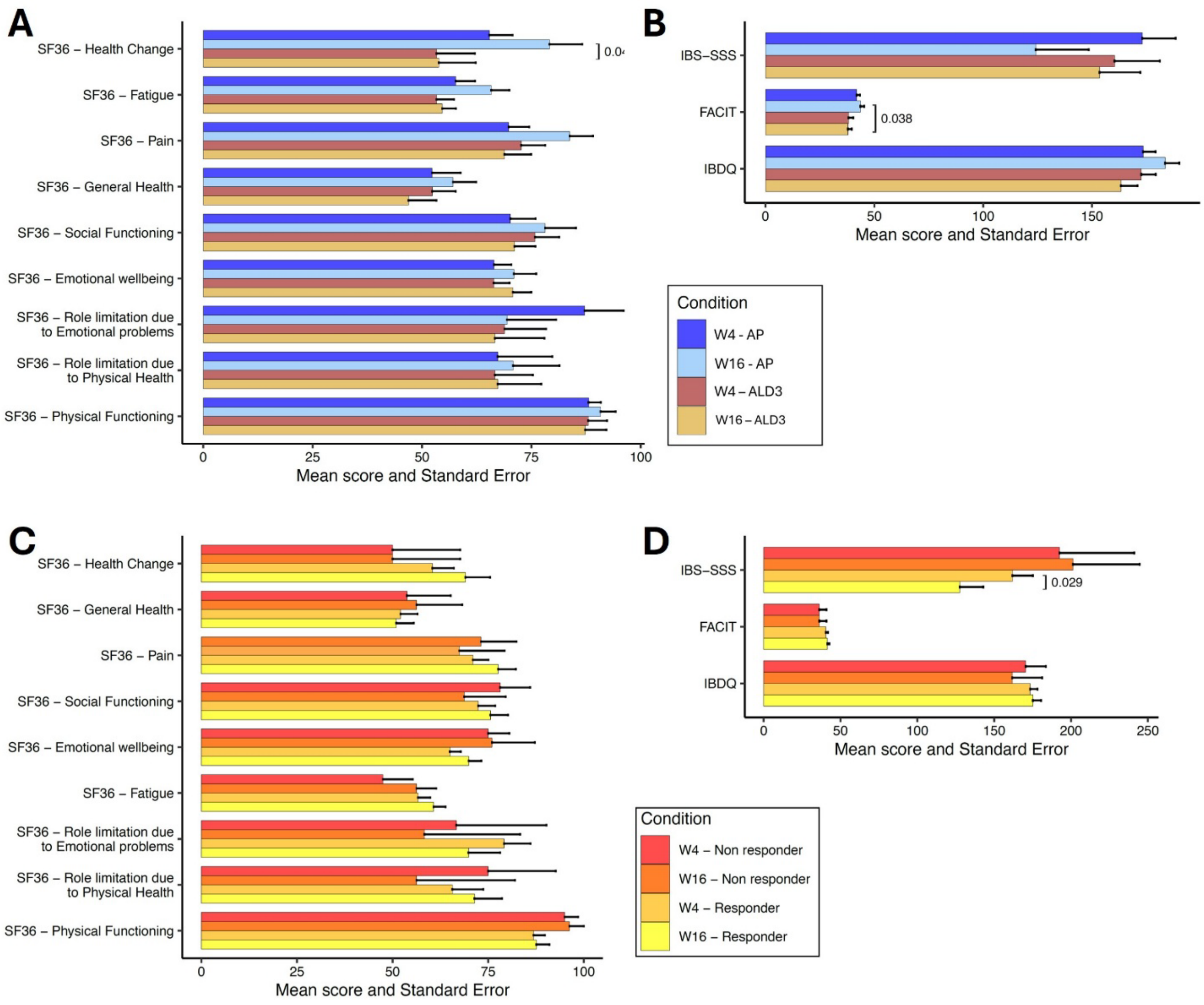

Supplementary Figure S2. Exploratory analysis of HRQoL and symptom questionnaires at week 16 (W16). *(A,B) Mean scores ± standard error of HRQoL and symptom-related questionnaires at week 4 (W4) and week 16 (W16) in placebo-treated (AP; dark and light blue bars) and LGG+VitD3-treated patients (ALD3; red and ocher bars). (C,D) Mean scores ± standard error at W4 and W16 stratified by clinical response status (non-responders: red/orange bars; responders: yellow shades). Statistical comparisons were performed using two-tailed Wilcoxon–Mann–Whitney tests. P < 0.05 was considered statistically significant.*

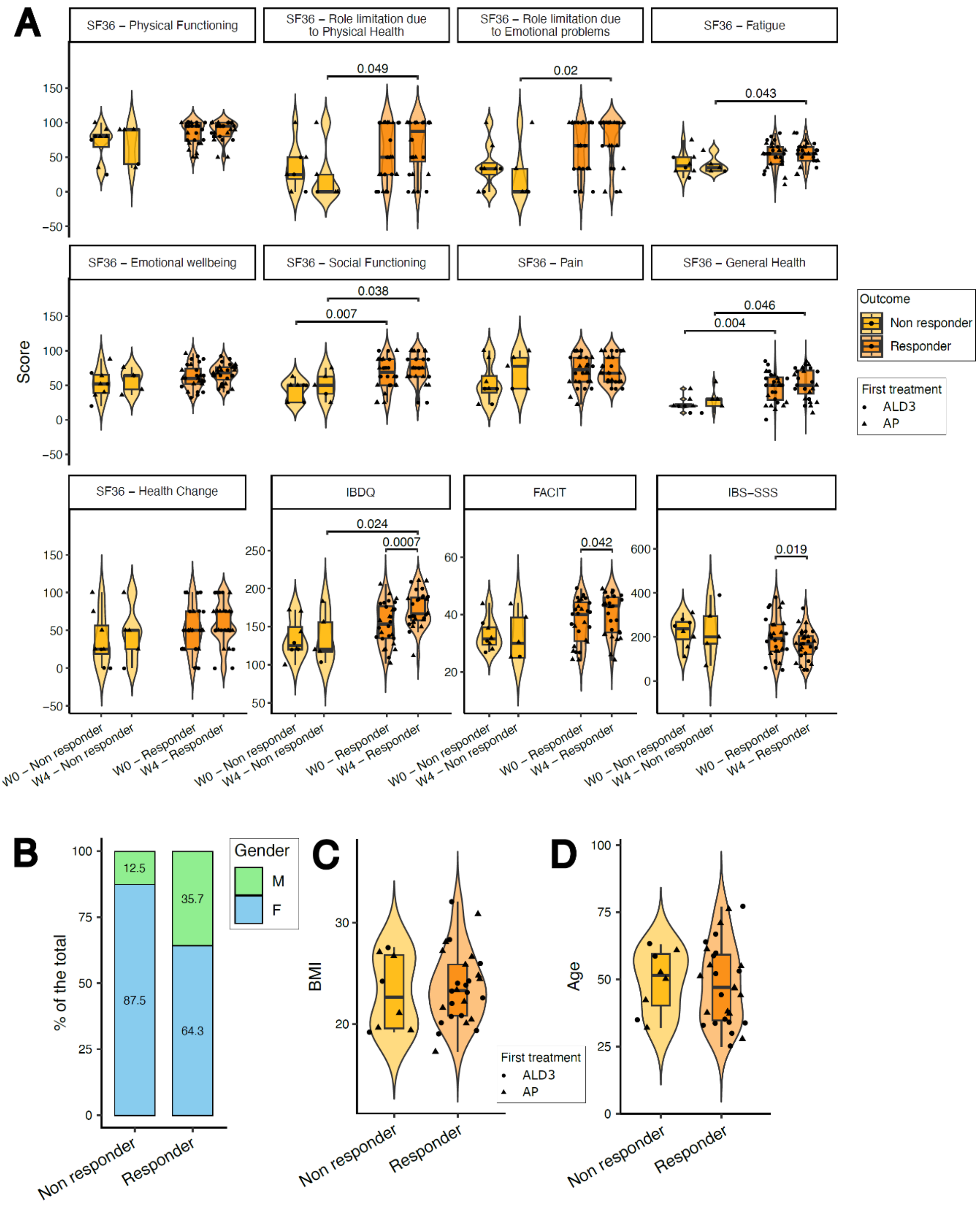

Supplementary Figure S3: Changes in patient-reported outcomes and clinical characteristics at week 4 stratified by response status. *HRQoL, BMI, and age in responders and non-responders after 4 weeks. (A) Score variation of the evaluated questionnaires at enrollment (W0), at the end of supplementation (W4) in clinical responders (orange graphs) or non-responder subjects (yellow graphs). Statistical analysis has been performed by Wilcoxon-Mann-Whitney test, two tailed. P<0.05 was considered statistically significant. (B) Percentage of males (light green) and females (light blue)*

in clinical responders and non-responders at W4. Statistical difference was evaluated with Fisher's exact test (p = 0.39). (C,D) Distribution of BMI (C) and age (D) between clinical responders (orange) and non-responders (yellow) at W4. Statistical analysis has been performed by Wilcoxon-Mann-Whitney test, two tailed.

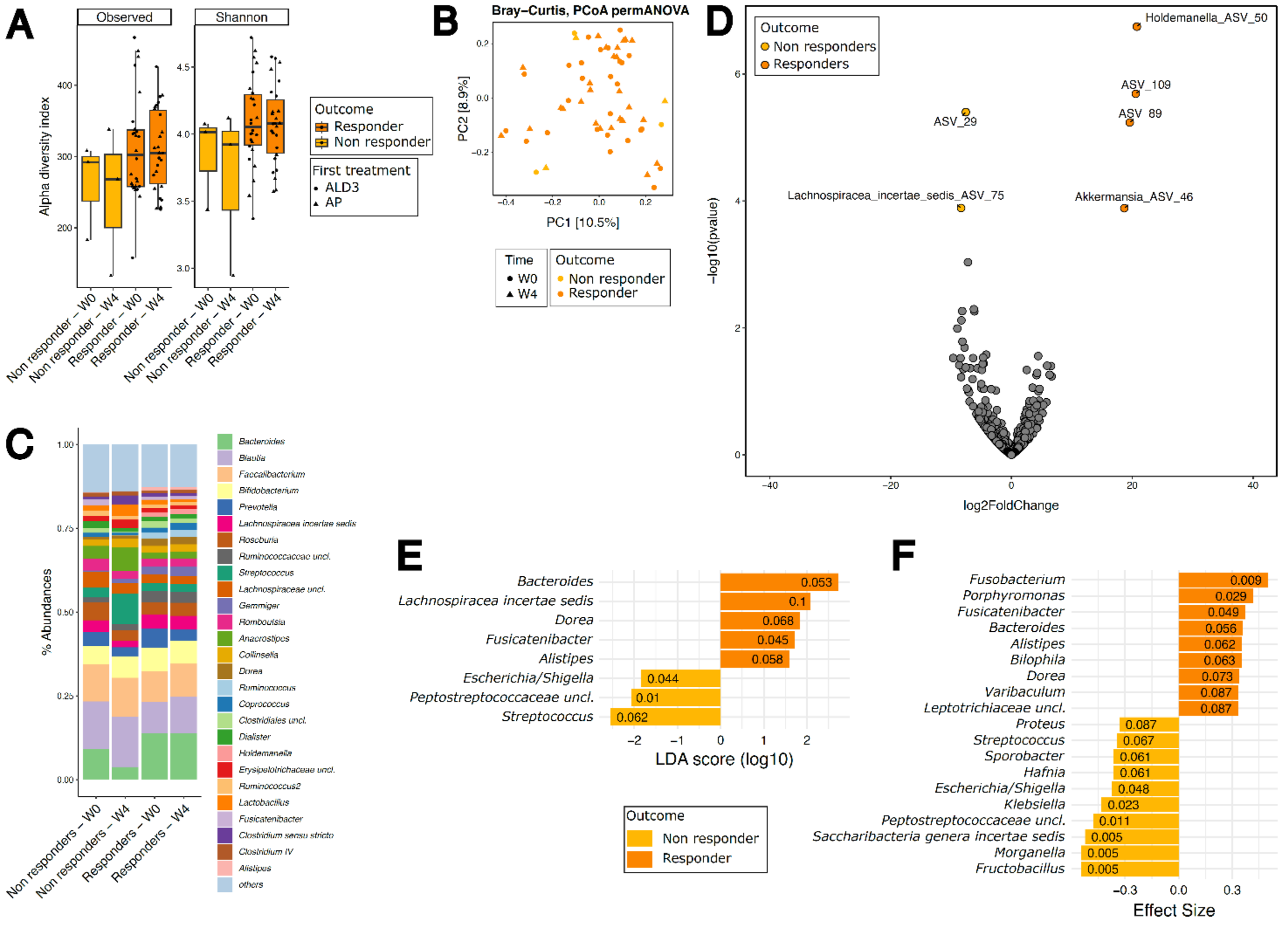

Supplementary Figure S4: Microbiome analyses at the end of W4 *(A,B) alpha (A, Observed and Shannon index) and beta (B, Bray-Curtis distance) diversity of non-responders (yellow symbols) and clinical responders (orange symbols) patients at baseline and W4. Statistical analysis on alpha diversity scores has been performed by Wilcoxon-Mann-Whitney test, two tailed. P<0.05 was considered statistically significant. Statistical analysis on beta diversity scores has been performed by PERMANOVA (not significant). (C) Mean relative abundance at W0 and W4 of the most abundant bacterial taxa in clinical responders and non-responders. (D) Volcano plot of the most significant enriched taxa after supplementation (W4) in non-responders (yellow symbols) and clinical responders (orange symbols) patients. Colored points are those with absolute log2FC > 1 and FDR*

< 0.05; name is reported for ASVs with absolute log2FC > 1 and FDR < 0.05. (E,F) LDA (E) and effect size (F) of the taxa enrichment at W4 between non-responders (yellow bars) and clinical responders (orange bars) patients. Statistical analysis has been performed by LEfSe and Mann-Whitney test, two tailed.
